# The binding study advice in medical education: a 2-year experience

**DOI:** 10.1007/s40037-015-0164-1

**Published:** 2015-02-05

**Authors:** Thijs M.H. Eijsvogels, Ronald Goorden, Wil van den Bosch, Maria T.E. Hopman

**Affiliations:** 1 Department of Physiology, Radboud University Medical Center, PO Box 9101, 6500 HB Nijmegen, The Netherlands; 2 Department of Cardiology, Hartford Hospital, Hartford, Connecticut USA; 3 Faculty of Medical Sciences, Radboud University Medical Center, Nijmegen, The Netherlands; 4 Department of Primary Care and Public Health, Radboud University Medical Center, Nijmegen, The Netherlands

**Keywords:** Binding study advice, Propaedeutic phase, Selection procedure, Medicine, Higher education

## Abstract

To improve the effectiveness of higher education, Dutch universities implemented the binding study advice at medical faculties. Accordingly, medicine students of Radboud University need to gain ≥ 42 out of 60 European *Credit* Transfer System (ECTS) credits to obtain a positive binding study advice and to continue their study programme. In case of a negative advice, the student is obliged to terminate the study, and he/she cannot register for the same study programme in the Netherlands within the next three years. The purpose of this manuscript is to evaluate the effect of implementation of the binding study advice on study outcomes. First, the binding study advice did not impact on student performance, as the average ECTS credits were comparable before and after its introduction. Second, study progress improved 8 % with 93 % of the students obtaining access to the second year of the study programme after binding study advice implementation. Third, the binding study advice did not impact propaedeutic graduation rates. These data demonstrate that the implementation of the binding study advice in medical faculties has only a small impact on study outcomes. The high performance levels of medical students compared with peers at other faculties are likely to contribute to these findings and suggest a ‘ceiling effect’ in the potential improvement of study outcomes at medical faculties.

Students who underperform or drop out in the first year of their study programme are a common phenomenon. In a Dutch cohort of 31,000 novice students, only 62 % were following the same programme after one year, while 28 % of the students switched to another programme and 10 % stopped studying all together [1]. The average graduation rate of 51 % after 7 years emphasises the inefficiency of higher education [2]. Therefore, the economic consequences for students and universities are substantial and countermeasures are necessary to improve its effectiveness.

A potential measure is the implementation of a binding study advice during the propaedeutic phase of a study programme. Dutch universities are allowed to provide students with a positive or negative binding study advice according to Article 7.8b of the Law on Higher Education and Scientific Research (*Wet op het Hoger Onderwijs en Wetenschappelijk Onderzoek*) [3]. The binding study advice depends on the number of European *Credit* Transfer System (ECTS) credits that a student collects during the propaedeutic phase. Universities are allowed to set the threshold value of ECTS credits for a positive or negative binding study advice themselves. A positive binding study advice allows a student to access the second year of the study programme. In contrast, a negative advice means an obligatory termination of the study, which means that the student cannot register for the same study programme in the Netherlands for a minimum of 3 years.

Over the past years, almost all Dutch universities have implemented the binding study advice at their medical faculties [4–10]. The underlying idea is that the binding study advice: I) stimulates the learning behaviour of students, II) excludes underperforming students in an early phase of the study programme, III) improves the graduation rates (preferably within a nominal timeframe), and IV) increases the return on investment rate of the university. The Radboud University introduced the binding study advice at the medical faculty in September 2011.

A student in the propaedeutic phase needs to earn ≥ 42 out of the 60 ECTS credits (a score of 70 %) to obtain a positive advice. The propaedeutic year of the Nijmegen curriculum consists of 10 courses of 4 weeks each, which all represent 5.5 ECTS credits. Moreover, the student needs to successfully complete the course Introduction into the Practice of Medicine (3 ECTS) and ≥ 3 of the Medical Progress Examinations (4 separate tests in September, December, February and May; 2 ECTS in total). An interim binding study advice is provided in February to warn students regarding their study performance and progress. Then, a provisional binding study advice is provided in the first week of August, allowing students with a negative advice to explain their underperformance. Students who have personal reasons (e.g. illness or family problems) for the low number of ECTS credits that they collected throughout the year may convince the binding study advice committee to change their advice by explaining and providing documentary evidence of their personal circumstances in a hearing (e.g. evidence of diagnosis). The final binding study advice is communicated in August. Although this is the final advice of the binding study advice committee, students have an ultimate possibility to start an official objection procedure within 6 weeks. The students who do object, do so as soon as possible because the next course starts in September and the negative advice does not allow them to participate.

Though the binding study advice is implemented at all Dutch medical faculties, there is currently no evidence available regarding its effect on study performance (collected ECTS credits), study progress (% granted access to year 2 of the study programme), and graduation rates (% obtained propaedeutic diploma). For this purpose we compared data from medical students who started the study of medicine at Radboud University Nijmegen between the academic years of 2008 and 2013. Data from the academic years 2008/2009, 2009/2010 and 2010/2011 were set as baseline values (no binding study advice), and compared with data from 2011/2012 and 2012/2013 (both after implementation of the binding study advice).

The number of students who were enrolled in the study programme was stable across the years (*n* = 328 ± 2). On average, students collected 53 ± 15–55 ± 12 ECTS credits in the control years, while this score did not change in the 2 years after implementation of the binding study advice (54 ± 12 and 55 ± 11 ECTS). In contrast, the number of students who collected enough credits to proceed to the second year increased from an average of 85 % in the control years to 92 and 93 % in the academic years of 2011/2012 and 2012/2013, respectively. Also, the graduation rates slightly increased from an average of 61 % prior to 66 % after the introduction of the binding study advice (Table 1).

**Table 1:** The impact of the implementation of the binding study advice (BSA) on study performance, study progress, and graduation rates

	Control years					Implementation of BSA	
	2008/2009		2009/2010		2010/2011	2011/2012	2012/2013
Started study program (*n* (%))	330 (100 %)		330 (100 %)		326 (100 %)	328 (100 %)	327 (100 %)
Stopped voluntarily before interim BSA (*n* (%))						7 (2 %)	5 (2 %)
*Interim BSA*							
Positive (*n* (%))						283 (86 %)	291 (89 %)
Negative (*n* (%))						38 (12 %)	31 (9 %)
*Provisional BSA*							
Positive (*n* (%))						305 (93 %)	302 (92 %)
Negative (*n* (%))						16 (5 %)	20 (6 %)
*Final BSA (n (%))*							
Positive (*n* (%))						302 (92 %)	305 (93 %)
Negative (*n* (%))						13 (4 %)	15 (5 %)
Objection (*n* (%))						2 (1 %)	1 (0 %)
Objection approved (*n* (%))						0 (0 %)	1 (0 %)
Stopped voluntarily throughout year (*n* (%))	10 (3 %)		12 (4 %)		9 (3 %)	13 (4 %)	7 (2 %)
Study performance (collected ECTS credits (mean ± SD))	53 ± 15		53 ± 15		55 ± 12	54 ± 12	55 ± 11
Study progress (granted access to year 2 (*n* (%))	280 (85 %)		266 (81 %)		289 (89 %)	302 (92 %)	305 (93 %)
Graduation rates propaedeutic phase (*n* (%))	211 (64 %)		172 (52 %)		222 (68 %)	222 (68 %)	212 (65 %)

*BSA* binding study advice, *ECTS* European Credit Transfer System

From a binding study advice perspective, 2 % of the students decided to stop their study before they received an interim advice in February. Of the remaining population, *n* = 38 (12 %) and *n* = 31 (9 %) received an interim negative binding study advice in 2011/2012 and 2012/2013, respectively. These numbers dropped to *n* = 16 (5 %) and *n* = 20 (6 %) for the provisional negative advice in the first week of August, and further decreased to *n* = 13 (4 %) and *n* = 15 (5 %) for a final negative binding study advice. Only three students decided to start an official objection procedure, with only one of them being successful. An overview of our findings is presented in Table 1.

The similar number of ECTS credits that were collected across the three academic years demonstrates that the implementation of binding study advice has no impact on the study performance of medical students. Although counterintuitive, these findings were superior compared with a study by Koning and colleagues who reported a decrease in collected ECTS credits (50 ± 18 *versus* 47 ± 20) after implementation of the binding study advice in a large cohort of psychology students (*n* = 961) at Erasmus University Rotterdam [11]. The authors suggested that students may aim for the binding study advice threshold of ECTS credits, rather than achieving an optimal study performance. Given the average score of 54 out of 60 ECTS credits (90 %), which is well above the ≥ 42 ECTS threshold, this explanation is unlikely to apply to medical students. Alternatively, we propose that a ‘ceiling effect’ in study performance may explain the lack of improvement in collected ECTS credits. As the average medical student collects 90 % of the available ECTS credits, it is almost impossible to further improve study performance in these students.

A second important finding of our study was the increase in study progress. The binding study advice caused a 8 % rise in students who collected enough ECTS credits to obtain access to the second year of the study programme (85 % *versus* 93 %), which is the equivalent of 29 students (*n* = 280 in control years *versus* 304 after implementation binding study advice). Interestingly, the difference in the number of students who received an interim negative advice (*n* = 31) and those who received a final negative advice (*n* = 15) may account for ~ 50 % (*n* = 16) of the improvement in study progress. We therefore hypothesize that the interim binding study advice acted as a kind of ‘wake-up call’ for these students, and resulted in improved study performance during the second half of the academic year. From this perspective, the implementation of the binding study advice resulted in the effect that was aimed for.

The academic years of 2011/2012 (68 %) and 2012/2013 (65 %) both demonstrated higher graduation rates compared with the 3-year average prior to introduction of the binding study advice (61 %). However, we must emphasize that this value was strongly influenced by the remarkably weak performance of the cohort of 2009/2010 (52 %). Excluding this cohort resulted in a comparable average graduation rate of the academic years pre/post introduction of the binding study advice (both 66 %). We therefore believe that the binding study advice does not have a large impact on propaedeutic graduation rates. Comparing the 2012/2013 propaedeutic graduation rates of medical students (65 %) with the graduation rates at the faculties of Science (35 %), Philosophy, Theology and Religious studies (40 %), Arts (47 %), Social Sciences (47 %), Law (48 %) and the Nijmegen School of Management (38 %) reveals that medical students perform significantly better compared with their peers at other faculties. These findings reinforce our ‘ceiling effect’ theory, as the optimal study performance of medical students is hard to improve by the binding study advice.

In conclusion, the implementation of the binding study advice at the medical faculty of the Radboud University was of low to moderate value in terms of study outcomes: medical students demonstrated improvements in study progress, while study performance and graduation rates were unaltered. The existing high performance levels amongst medical students prior to introduction of the binding study advice are likely to contribute to these findings and suggest a ‘ceiling effect’. Nevertheless, the binding study advice may still be useful to exclude underperforming students in an early stage of the study programme, which could contribute to improvement of the efficiency and reduction of the costs of medical education.

## Disclosure statement funding

Dr. Eijsvogels is financially supported by the Netherlands Organization for Scientific Research (Rubicon Grant 825.12.016).
